# Formaldehyde1Read at the thirty-third annual meeting of the Medical Association of Central New York, held at Rochester, N. Y., October 16, 1900.

**Published:** 1901-03

**Authors:** A. A. Young

**Affiliations:** Newark, N. Y.


### FORMALDEHYDE.[1]

By A. A. YOUNG, M. D., Newark, N. Y.

FORMIC aldehyde, an oxide of methylene, is a gaseous substance, the molecular composition of which is C H2 O, or one atom of carbon, and one atom of oxygen united by two atoms of hydrogen in each molecule. It is readily soluble in water, producing about a 40 per cent. solution which borders upon saturation, and in this form it is found as an article of commerce. Upon evaporation of the solution beyond the point of saturation a re-arrangement of the atoms takes place within the molecule, the composition of which is now $C_3H_6O_3$, known as paraform, a white crystalline substance, which it will be observed, forms a molecule of twelve atoms instead of four atoms, as in the case of the gaseous substance. It is a well established fact in chemistry that the re-arrangement of atoms within the molecule produces an entirely different mass with entirely different characteristics and properties, notable examples of which are oxygen and ozone, or the diamond plumbago and charcoal. It is for this reason, I believe, without experimentation, that in this polymeric modification paraform loses in the transformation nearly all the properties of formaldehyde, including its preservative and germicidal actions.

---

1. Read at the thirty-third annual meeting of the Medical Association of Central New York, held at Rochester, N. Y., October 16, 1900.

In this connection it will be observed that when paraform is heated in an open vessel it vaporises, but the vapor on cooling deposits, as before, crystalline paraform by sublimation; but when formaldehyde is heated in an open vessel, and much less heat is required, formic aldehyde the germicidal agent, is given off vaporised, and so far as known is not redeposited as paraform; but upon the sides of the container, by slow evaporation, this polymeric modification is found deposited. This phenomenon indicates most clearly a chemical change; a change which means new properties and new characteristics; experimentation, so far as made, indicates the truth of this assumption. Paraform is, therefore, regarded by the writer as a by-product, a new formation, and cannot consequently be compared with formaldehyde.

They are not identical substances, though one may be formed from the other by synthesis or analysis. To convert one into the other requires a chemical change, a re-arrangement of the atoms within the molecule; it means a new chemical compound having entirely different characteristics and actions. Recognising this fact, it must be apparent to all that the properties of the one cannot be substituted for the properties of the other by inference, for the indications are that error must certainly follow as the result.

It is a mistaken notion that if formaldehyde be a germicidal agent, paraform, its by-product, as well must be one.

In this paper, therefore, only formic aldehyde, or its liquid solution, as found in commerce, known as formaldehyde (Merck), will be referred to.

Although formaldehyde was discovered as early as 1863, but little was known of its properties until recent years, its use being entirely confined to the microscopist, who employed it as a hardening and preserving agent. In its actions upon the tissues but few changes are observable, the tissues simply harden without material shrinking or discoloration.

In the specimens I herewith present, you will observe that the outlines in each are clear and strong, that there is no shrinking in any part, that each specimen is more firm and hard than found in its natural state, and that the natural colors are not affected by the agent.

If, then, formaldehyde possesses such marked properties as a preservative and hardening agent, in like manner must it be, according to theory, a germicidal agent as well, and experimentation testifies to the truth of the assumption. It has been found by experimentation that solutions as attenuated as 1 to 100,000 destroy quite readily the less resistant germs, while the more resistant ones are readily destroyed when immersed in a solution of 1 to 1,000. In this connection it should be observed that in solutions of the latter strength even, that the living human skin (example, the hands), is not appreciably affected by such solution, though the hands may remain in the liquid for a considerable time.

Although formaldehyde possesses such marked hardening properties it exerts no particular influence over mucus or albumen, save to render these substances aseptic or rather antiseptic, a condition that may be advantageous in its therapeutic uses. Of its specific action but little is known, save that it is, of most organic tissues, a most excellent hardening agent, and a germicide of the highest potency. Accepting, then, the theory that most of our diseases, especially the communicable ones, are the result of the action or growth of specific bacteria, and that the destruction of specific bacteria, in situ or elsewhere, stands for prophylaxis, alleviation or cure of such specific disease, and for which bacteria stands as the index, then the therapeutic use of formaldehyde, to me at least, is a suggestive one, and, clinically, in several directions I have experimented along this line with most favorable results, which results it is my purpose briefly to give.

The gas when liberated has the properties of diffusibility and penetrability in a marked degree, and therefore it is admirably adapted for disinfecting purposes, especially small rooms and exposed objects therein; but too great results must not be expected of it, for it will not permeate impermeable objects, and because of this failure it should not be condemned, though some writers have so condemned it. In passing, suffice it to say its penetrability, its effectiveness as a germicide, its comparative harmlessness upon objects with which it comes in contact, indicates it as worthy of an extended experimentation along therapeutic lines. Its use in this relation, as it has occurred in my practice will now be briefly given.

(*a*) Over four years ago Mr. C. sought advice for a diseased testicle, which I then believed to be malignant; its removal was advised and an operation performed with the use of mercuric bichloride as an antiseptic; recovery was much retarded by the formation of a fistula beginning near the upper border of the scrotum and passing upward along the tract of the cord; from this fistula there was constant discharge. This fistula was frequently cleansed with peroxide of hydrogen and followed with various other germicides, but without material improvement. A change in the method of treatment seemed desirable. I then placed upon a small probe a bit of cotton, which was just nicely moistened with water; immersing this cotton into full strength formaldehyde and removing the superfluous agent from the cotton by a couple of swings of the probe in the air; it was then inserted into and passed full length of the sinus, the smarting sensation was severe for a moment only. Suffice it to say, repair began at once and within one week there was full restoration of the parts affected.

About five months later the opposite testicle became similarly affected. For this operation formaldehyde was exclusively used in place of mercuric bichloride, and in the subsequent lavage and dressings the use of the agent was continued. Recovery was rapid without discharge or formation of a sinus. The patient is still living and healthful, with no indications of any malignant growth anywhere about the body.

For operative procedures in case of ruptured perineum, either recent or old, formaldehyde has been used as a cleansing agent with pleasing results; it coagulates the exudates, hardens the ruptured tissues with which it comes in contact, forming a barrier against septic infection from without, and as such hardening agent it proves to be a most excellent hemostatic as well. I have used it several times, more especially in cases immediately following confinement. Such wounds, rough and ragged as they might be, invariably repaired by first intention and without septic infection, a condition doubtless brought about by the peculiar action of this agent directly upon the lacerated tissues and exudates from such laceration.

(*b*) About three years ago Mr. D. complained of pain in and about the rectum. Examination revealed an anal fistula, blind external. The usual operation was performed and the wound curetted after which full strength formaldehyde was applied to the entire raw surface; the wound was well cleansed and partially closed in order to retain more fully the normal mechanical action of the sphincter ani. The external dressings were moistened with a weak solution of formaldehyde; restoration of the parts was rapid, reaching practically a perfect result.

(*c*) About three years ago Mr. V. requested treatment for bromidrosis; the case was a most severe one and various remedies had been tried, but without material benefit. A 10 per cent. solution of formaldehyde was prescribed, which checked sweating at once, and following this some ten days later there was exfoliation of the affected skin, leaving a healthful skin underneath. So far as known there has been no return of the affection.

(*d*) Various growths and pathological conditions of the skin have readily been destroyed by this agent, notably indolent ulcers, especially of the lip and face; molluscum contagiosum and allied affections by applications of formaldehyde have readily lost their vitality, then cleaving from the healthful skin, thus making their destruction complete.

Not alone in surgical work is the use of this agent indicated, but from a medical point of view, its peculiar properties suggest its use and in accordance with such suggestion it has been guardedly administered and its general actions noted as far as possible:

(*a*) Over four years ago Miss M., a dressmaker, began to complain of a gastric disturbance, the signs and symptoms of which were indicative of gastric ulcer. This condition existed in a varying degree of severity till about one year ago, when severe hemorrhage occurred, severe enough to threaten life. Ordinary remedies were administered and hemorrhage largely, but not entirely, controlled and there remained occasional attacks of vomiting, which showed degenerative changes within the stomach. Improvement was not satisfactory. Believing that this condition was induced by the development of some microörganisms, or the activity of some of the digestive ferments within the stomach, pure antiseptic treatment was decided upon and formaldehyde chosen as the agent.

Two minium doses were given every four hours well diluted. Immediately following its administration satisfactory and rapid improvement was noted, and the lady is now enjoying better health than she has enjoyed for years. The mode of administration, which was most satisfactory to the patient as well as to myself, was to dilute two minims of the agent in two drachms of water, swallow quickly this portion and follow it at once by half a glassful of water; the smarting sensation was then reduced to a minimum.

(*b*) About two months ago I was summoned to attend a case of dysenteric diarrhea in a child. The case was a very severe one from the onset; the child grew gradually worse until death was hourly expected; a change was then made from the usual remedies and formaldehyde was prescribed in one minim doses, mixed with egg-albumen; the disease in its active stage was soon arrested and his life prolonged, but the integrity of the intestinal tract was so altered that death occurred some ten days later, due to malnutrition.

A younger child in the same family was similarly affected and not till formaldehyde was prescribed, as in the case of the boy, were changes apparent; the child then made a rapid recovery.

Lastly, your attention is called to formaldehyde as a prophylactic agent. In the eruptive diseases, more especially so in scarlet fever —on account of the hardening and germicidal properties of this agent —is its value most apparent.

We are all aware how hard it is to enforce strict quarantine over a patient during the entire period of desquamation; we are also aware how difficult it is to determine when no danger exists, when no specific microörganisms can be conveyed from the convalescent to the uninffected. Experimentation has proven, to my satisfaction at least, that when it is applied externally, in scarlatinal cases, beginning with the eruptive stage, and carried well into the desquamative stage, the latter stage is materially shortened, while the vitality of these microörganisms is destroyed within the nidus, the scale, a considerable period of time before desquamation is completed; this conclusion is based upon experience, an experience that warrants the opinion that as such prophylactic agent, it stands without a peer.

It matters not, if the germ be dead, where the scale may go, it can do no harm, it can produce no life, its power of reproduction is destroyed; reinfection must come from a new source.

Its use, as a medicinal agent, is only advocated where germicidal medication is called for and indicated; not that it will cure (?) all diseases all of the time, but rather that it may affect, in a desirable way, some of the specific diseases a part of the time.

22 East Miller Street.

